# Confusion and Conflict in Assessing the Physical Activity Status of Middle-Aged Men

**DOI:** 10.1371/journal.pone.0004337

**Published:** 2009-02-02

**Authors:** Dylan Thompson, Alan M. Batterham, Daniella Markovitch, Natalie C. Dixon, Adam J. S. Lund, Jean-Philippe Walhin

**Affiliations:** 1 School for Health, University of Bath, Bath, United Kingdom; 2 Health and Social Care Institute, University of Teesside, Middlesbrough, United Kingdom; Dalhousie University, Canada

## Abstract

**Background:**

Physical activity (including exercise) is prescribed for health and there are various recommendations that can be used to gauge physical activity status. The objective of the current study was to determine whether twelve commonly-used physical activity recommendations similarly classified middle-aged men as sufficiently active for general health.

**Methods and Findings:**

We examined the commonality in the classification of physical activity status between twelve variations of physical activity recommendations for general health in ninety men aged 45–64 years. Physical activity was assessed using synchronised accelerometry and heart rate.

Using different guidelines but the same raw data, the proportion of men defined as active ranged from to 11% to 98% for individual recommendations (median 73%, IQR 30% to 87%). There was very poor absolute agreement between the recommendations, with an intraclass correlation coefficient (A,1) of 0.24 (95% CI, 0.15 to 0.34). Only 8% of men met all 12 recommendations and would therefore be unanimously classified as active and only one man failed to meet every recommendation and would therefore be unanimously classified as not sufficiently active. The wide variability in physical activity classification was explained by ostensibly subtle differences between the 12 recommendations for thresholds related to activity volume (time or energy), distribution (e.g., number of days of the week), moderate intensity cut-point (e.g., 3 *vs.* 4 metabolic equivalents or METs), and duration (including bout length).

**Conclusions:**

Physical activity status varies enormously depending on the physical activity recommendation that is applied and even ostensibly small differences have a major impact. Approximately nine out of every ten men in the present study could be variably described as either active or not sufficiently active. Either the effective dose or prescription that underlies each physical activity recommendation is different or each recommendation is seeking the same prescriptive outcome but with variable success.

## Introduction

There has been progressive improvement in our understanding of the role of physical activity in the protection against non-communicable chronic diseases such as cardiovascular disease [1]–[3]. The accumulated evidence has been used to develop guidelines for the recommended minimum level of physical activity to promote and maintain general health [1]–[8] and these guidelines are used to describe the relative prevalence of ‘inactivity’ or ‘sedentarism’ in defined populations [9], [10]. At an individual patient level, healthcare practitioners are encouraged to view exercise as medicine [11] and recent public health intervention guidance from the National Institute for Health and Clinical Excellence (NICE) in the UK recommend that primary care practitioners should take every opportunity to identify inactive adults [12]. Healthcare practitioners rely on and are encouraged to use physical activity recommendations to gauge a patient's current behaviour in answer to the logical question ‘am I doing enough physical activity for health?’ [12], [13].

Physical activity recommendations attempt to deliver a clear and unambiguous public health message about the appropriate ‘dose’ of physical activity to promote and maintain health. Usually, recommendations are expressed as the requirement to either undertake a certain amount of physical activity above specific intensity thresholds (e.g., time engaged in moderate intensity activity on a certain number of days per week) or to undertake a specified volume of physical activity (e.g., overall physical activity energy expenditure). It is only recently that suitably accurate and precise measurement tools have become available to simultaneously capture both free-living physical activity volume and pattern over a sufficiently long period of time to allow comparisons between physical activity recommendations [14]–[17].

Physical activity recommendations usually reflect the *minimum* dose required for general health or the point at which people accumulate substantial health benefit [1], [3]–[6], [8] Clearly, because recommendations are seen as the lower limit of acceptable physical activity, it is imperative for individuals and healthcare practitioners that such judgments are consistent and unambiguous. In August 2007, new physical activity recommendations were jointly released by the American College of Sports Medicine (ACSM) and the American Heart Association (AHA) [5] to replace earlier recommendations [1]. In October 2008, the US Department of Health and Human Services (USDHHS) released new physical activity recommendations [8] and these have been adopted and promoted by the Centers for Disease Control in the US [18]. These recommendations sit alongside a variety of recommendations such as those issued by the Chief Medical Officer in the UK [3] and the Institute of Medicine in the US [4]. On the surface, physical activity recommendations appear very similar but we sought to examine whether ostensibly subtle differences between recommendations influenced physical activity status.

## Methods

### Participants

Following local ethics approval, we recruited men aged 45–64 years from the local community by advertisement in local newspapers and bulletin boards in public places. In order to take part, volunteers had to be asymptomatic non-smokers who were not taking medication and who had a Body Mass Index (BMI) ≤35 kg/m^2^. Mean age, height, body mass and BMI were 53±5 years (range 45–63), 1.80±0.06 m (range 1.64–1.96), 88±11 kg (range 63–130) and 27±3 kg/m^2^ (range 21–34).

### Assessment of Physical Activity Energy Expenditure

Physical activity energy expenditure was estimated using synchronized accelerometry and heart rate with branched equation-modelling (Actiheart, Cambridge Neurotechnology Ltd., Cambridge, UK) as previously described [13], [15]. This technique provides accurate and precise estimates of energy expenditure during physical activity [14]–[17]. Participants wore this instrument for nine continuous days and data were logged for every minute throughout this period (day and night). Data from the first and last day were discarded as this was not a full 24 h period and therefore results represent seven full days of observation. Participants were instructed to only remove the physical activity monitor to change the electrodes. If there was more than a 15 min period in any given day where there was no heart rate signal (either due to non-compliance or technical difficulties) we automatically excluded this participant from the analysis.

Many physical activity recommendations refer to physical activity intensity expressed in Metabolic Equivalents (METs) where 1 MET represents resting oxygen consumption assumed to equal 3.5 ml O_2_/kg/min. In order to convert energy expenditure in kcal/min to METs we used age-specific equations for Basal Energy Expenditure (BEE) [19]. Physical activity energy expenditure was added to basal energy expenditure to provide total energy expenditure (with basal energy expenditure expressed per minute assumed to equal 1 MET). In-house software was developed to examine the number of minutes engaged in physical activity above different intensity thresholds (e.g., 3×BEE or 3 METs) and different durations (e.g., 10 min). This allowed us to quantify the total amount of time engaged in physical activity above specific thresholds on either a minute-by-minute basis or in specific bouts (e.g., 10 min bouts above 3 METs).

### Physical Activity Recommendations

For this analysis we examined the ACSM/CDC and US Surgeon General recommendations that have been widely adopted worldwide for the past ten years [1], [6], the current recommendations from ACSM/AHA [5], the current recommendation from USDHHS [8] that are endorsed by CDC [18], the current recommendations from the UK Chief Medical Officer and Department of Health (DoH) [3], and the current recommendations from the US Institute of Medicine [4]. For some recommendations there was imprecision in expression which meant that various interpretations were possible for the same recommendation. Occasionally, recommendations were expressed using different outcomes (e.g., time engaged in moderate intensity physical activity *vs.* energy expenditure). Where relevant and appropriate, we have included in our analysis the presentation of different outcomes and interpretations for each recommendation (Table 1).

**Table 1:** A summary of the physical activity recommendations and definitions that were assessed in the present comparison.

Recommendation	Definition
ACSM/CDC^1^	30 min of moderate intensity physical activity (≥3 METs) accumulated on a minute-by-minute basis on at least 4 days a week
CDC^1^	30 min of moderate intensity physical activity (≥4 METs) accumulated on a minute-by-minute basis on at least 4 days a week
ACSM/CDC^2^	30 min of moderate intensity physical activity (≥3 METs) accumulated in at least 10 min bouts on at least 4 days a week
CDC^2^	30 min of moderate intensity physical activity (≥4 METs) accumulated in at least 10 min bouts on at least 4 days a week
ACSM/AHA^1^	30 min of moderate intensity activity (3–6 METs) on at least 5 days in bouts of 10 min or 20 min of vigorous intensity activity (≥6 METs) on at least 3 days in bouts of 10 min
ACSM/AHA^2^	450 MET·min·wk^−1^ in discrete 10 min bouts of moderate (3–6 METs) or vigorous (≥6 METs) intensity activity (without taking into account either the number or distribution of these bouts throughout the week)
ACSM/AHA^3^	450 MET·min·wk^−1^ in discrete 10 min bouts of moderate or vigorous intensity activity and in at least the minimum number of ‘required’ bouts (i.e., 100% of either the moderate, vigorous or combined recommendation in terms of the 450 MET·min·wk^−1^ target, time and number of bouts), but without taking into account whether bouts were distributed across a specified number of days
DoH	30 min of moderate intensity physical activity (≥3 METs) accumulated in at least 10 min bouts on at least 5 days a week
IOM^1^	A Physical Activity Level (PAL; TEE/BEE) ≥1.6
IOM^2^	An average of ≥60 minutes of moderate intensity physical activity (≥3 METs) per day
USDHHS/CDC^1^	150 min of moderate intensity physical activity (3–6 METs) or 75 min of vigorous intensity activity (≥6 METs) per week in bouts of at least 10 min; or a proportional combination of moderate and vigorous intensity activity to meet a combined target.
USDHHS/CDC^2^	500 MET·min·wk^−1^ above ≥3 METs in bouts of 10 min or more *

* We have assumed that the requirement to undertake activity is >3 METs and in bouts of 10 min because this is a feature of the ‘time’ expression of this recommendation. This is not explicit but we felt that any other interpretation would be inappropriate (see methods for details).

### ACSM/CDC and CDC (1995, 1996)

These recommendations state that adults should accumulate 30 min or more of moderate-intensity physical activity on most, preferably all days of the week [1], [6]. Moderate intensity physical activity was defined as ≥3 METs in 1995 [6] for all age groups whereas in the report by the US Surgeon General from CDC moderate intensity physical activity for men aged 40–64 years was defined as 4.5–5.9 METs [1]. In a later position stand, moderate intensity physical activity for men aged 40–64 years was described as 4.0–5.9 METs [20]. Bout length was not specified in the 1995 recommendation [6] but the emphasis was on ‘accumulated’ activity and activities such as taking the stairs were suggested as acceptable. Therefore one interpretation is that *all* physical activity above 3 METs should be counted towards the 30-min target. On other occasions it was indicated that accumulation should be in *bouts* of 8–10 min in order to be counted [6]. In the report from the US Surgeon General, it was suggested that it was reasonable to expect the health benefits from accumulated short bouts (e.g., 10 min) to be the same as continuous activity [1]. There is clearly scope for numerous different interpretations of these recommendations but we limited our investigation to four different permutations (1) ACSM/CDC^1^ [6]; (2) CDC^1^ [1]; (3) ACSM/CDC^2^ [6]; and (4) CDC^2^ [1] as summarized in Table 1.

### ACSM/AHA (2007)

The current recommendation from ACSM/AHA [5] states that adults should either (A) accumulate a total of at least 30 min moderate intensity physical activity (≥3 METs) a day, five days a week (either in one session or in bouts of 10 min or more), or (B) engage in vigorous intensity activity (≥6 METs) three or more days per week for 20 min (either in one session or in bouts of 10 min or more). It is stated that a combination of moderate intensity and vigorous intensity physical activity can be used to meet this recommendation. It is proposed that the ‘credit’ for such activity could be assessed using the concept of MET·min (MET×min) and that 450 MET·min·wk^−1^ represents the minimum target. However, it is not clear whether the combination approach implies that 450 MET·min·wk^−1^ should be seen as the real target and it is less important that the activity is spread across a given minimum number of days (e.g., 3 or 5 days). One interpretation might be that it is important to meet the 450 MET·min·wk^−1^ target over a minimum number of discrete bouts of at least 10 min (presumably these being proportional to the earlier element of the recommendation; e.g., 30 min of moderate intensity physical activity five days a week in bouts ≥10 min) or simply that the combined total of moderate and vigorous intensity physical activity should equal 100% of the recommendation (e.g., 67% of the moderate recommendation and 33% of the vigorous recommendation). Clearly, there are numerous possible permutations but for the purpose of the current analysis we opted for three comparisons; (1) ACSM/AHA^1^ (2) ACSM/AHA^2^ and (3) ACSM/AHA^3^ as summarized in Table 1.

### Department of Health (2004)

The UK Chief Medical Officer and Department of Health recommend that for general health benefit adults should accumulate a total of at least 30 min moderate intensity activity (≥3 METs) a day, five days a week, either in one session or in bouts of 10 min or more [3].

### Institute of Medicine (2005)

The US Institute of Medicine (IOM) includes physical activity recommendations as part of its dietary recommendations [4]. The recommended level of physical activity is described as a Physical Activity Level or PAL (Total Energy Expenditure/Basal Energy Expenditure) greater than 1.6, which was estimated to be equivalent to ≥60 min of moderate intensity physical activity per day (where moderate intensity activities were ≥3 METs). The 60 min per day recommendation was based on the fact that, on average, 60 min of moderate intensity activity per day would be required to raise an individual from the ‘sedentary’ (PAL <1.39) to the ‘active’ category (PAL ≥1.6)[2]. We have examined both expressions of this recommendation as summarized in Table 1.

### USDHHS and CDC (2008)

The US Department of Health and Human Services (USDHHS) recommends that adults should do at least 150 min of moderate intensity physical activity (≥3 METs) or 75 min of vigorous intensity physical activity (≥6 METs) a week; or an equivalent combination of moderate and vigorous intensity activity [8]. The CDC has adopted this latest recommendation [18]. There is an explicit statement that the activity should be performed in bouts of at least 10 min. It is noteworthy that the recommendation expressed in weekly minutes of physical activity was reported to be derived from evidence on estimated energy expenditure with the intent being to ensure that people undertake at least 500 MET·min·wk^−1^. It is not explained whether the 500 MET·min·wk^−1^ target refers to energy expended above a certain physical activity intensity or duration threshold but, for the purpose of the current analysis, we have assumed that this is the case since otherwise this target would be very low and readily achievable (e.g., simply standing for 45 min a day without any other movement would comfortably exceed 500 MET^.^min at the end of a week; 1.8 METs * 45 * 7 = 567 METs). As a result, we have assumed that physical activity must be at least 3 METs to be counted and we have also assumed that it must be in bouts of at least 10 min. It is stated that ‘preferably’ physical activity should be distributed throughout the week – although this does not appear to be an explicit part of the recommendation and, if it is, it is not clear what the distribution of the activity should be. As a result, we have included just two permutations of this recommendation (using time or MET·min·wk^−1^) as shown in Table 1.

### Data Analysis

The main outcome variable is dichotomous – a participant either meets or fails to meet a specific recommendation – we calculated the proportion of the sample classified as physically active according to each. The median and interquartile range of these 12 proportions was calculated to illustrate the variability in the prevalence of sufficient physical activity. We considered it inappropriate to conduct pair-wise statistical comparisons of the 12 proportions (n = 66 pairs). Rather, we assessed the agreement between the 12 recommendations using the most appropriate 2-way, mixed-model form of the intraclass correlation coefficient (ICC, A,1; absolute agreement definition) [21]. The formula for this coefficient includes the between-recommendation (column) variance in the denominator, and is broadly equivalent to an average Kappa statistic. Adopting a model that includes column variance is essential, as our research question requires the evaluation of systematic differences in prevalence estimates between recommendations. Our sample size requirement was based on securing both adequate precision and representativeness. We pre-specified a small effect size of an ICC of 0.20, given by the mid-point of the category representing poor agreement for the Kappa statistic (0.00 to 0.39)[22]. A sample size of approximately 90 subjects was necessary to provide a 95% confidence interval width of 0.2 units [21] sufficient to differentiate the correlation from the lower boundary of the category representing fair to good agreement (0.40)[22]. Originally, this estimation was based on a comparison of 10 physical activity recommendations. Whilst this manuscript was undergoing review, however, a new guideline was released (USDHHS and CDC, 2008) adding two further recommendations. The addition of these recommendations does not alter our precision of estimation of the ICC appreciably, and the original sample size estimation stands. Twenty four subjects were excluded from the analysis for failing to provide complete data; recruitment and testing continued until 90 full, clean data sets were obtained. All data analyses were conducted using SPSS v.14, SPSS Inc., Chicago, IL).

## Results

### Physical activity (weekly average)

Subjects spent on average 124±49 min and 46±24 min a day engaged in physical activity above 3 METs and 4 METs, respectively (Table 2). Every subject accumulated on average more than 30 min of activity above 3 METs per day whereas 63 subjects (70%) performed on average more than 30 min of activity per day above 4 METs. On average, 66% of the subjects engaged in 30 min of activity or more per day above 3 METs in bouts of 10 min, whereas only 17% of the subjects engaged in 30 min of activity or more per day on average above 4 METs in bouts of 10 min. Every subject accumulated more than 150 min of physical activity a week above 3 METs when assessed on a minute-by-minute basis (minimum was 245 min) and 88 subjects (98%) performed more than 150 min of physical activity a week above 3 METs when assessed in bouts of 10 min or more.

**Table 2:** Mean time per day engaged in physical activity above critical thresholds for men aged 45–64 (n = 90).

	≥3 METs	≥4 METs	≥6 METs	≥3 METs^10^	≥4 METs^10^	≥6 METs^10^
Mean±SD	124±49	46±24	8±11	42±26	16±15	4±9
Range	35–336	5–132	0–53	0–132	0–65	0–37

METs^10^ represents time engaged in activity above each respective threshold in bouts of at least 10 min.

Estimated mean daily physical activity energy expenditure was 1083±337 kcal (range 596–2122) with 592±171 kcal being expended below 3 METs (range 239–1013), 416±185 kcal between 3 and 6 METs (range 116–1114) and 75±104 kcal above 6 METs (range 362–1731).

### Physical activity recommendations

**Figure 1:**
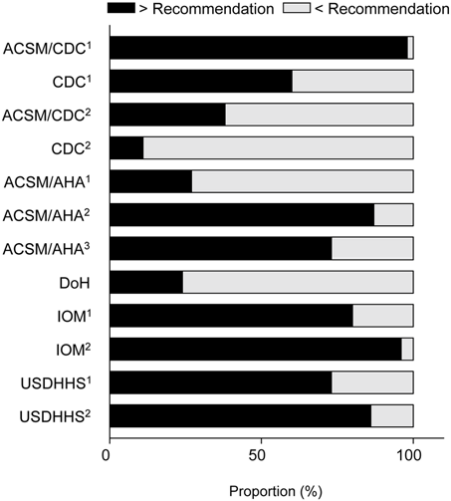
The proportion of middle-aged men in this sample who either met or failed to meet each of the 10 recommendations (n = 90).

The intraclass correlation coefficient (A,1) for the classification of individual subjects as active or not sufficiently active across the 12 recommendations was 0.24 (95% confidence interval, 0.15 to 0.34), indicating very poor absolute agreement. Agreement between prevalence estimates within separate clusters of recommendations forming the upper and lower tertiles of the distribution was also evaluated. The ICC (A, 1) for the 4 recommendations resulting in the lowest prevalence estimates (ACSM/CDC^2^, CDC^2^, ACSM/AHA^1^, and DoH) was 0.60 (0.49 to 0.71). For the cluster of 4 recommendations forming the upper tertile for prevalence (ACSM/CDC^1^, ACSM/AHA^2^, IOM^2^, and USDHHS/CDC^2^) the ICC (A, 1) was 0.46 (0.35 to 0.57). The median proportion (interquartile range) of men defined as sufficiently active across all recommendations was 73% (30% to 87%), with a range of 11% (CDC^2^) to 98% (ACSM/CDC^1^) for individual recommendations (Figure 1). Only 7 men (8%) met all 12 recommendations and would therefore unanimously be categorized as ‘active’ irrespective of the recommendation or interpretation being considered or applied. Only one subject failed to meet at least one recommendation and would therefore be unanimously categorized as ‘not sufficiently active’.

The difference between ACSM/CDC^1^ and CDC^1^ is the physical activity threshold of 3 *vs.* 4 METs, respectively. ACSM/CDC^1^ differs from ACSM/CDC^2^ in that for the latter only physical activity undertaken in bouts of at least 10 min contributes to accumulated physical activity. The only difference between ACSM/CDC^2^ and DoH is that activity should be undertaken on either most (i.e., 4 days) or 5 days a week, respectively. ACSM/AHA^1^ only differs from DoH in that vigorous activity can be used to replace moderate intensity physical activity over a reduced timescale. The small 50 MET·min·wk^−1^ difference between ACSM/AHA^2^ and USDHHS^2^ affected the classification of only one man in the present sample. Collectively, the differences between recommendations exert from small to very pronounced effects on the proportion of men that meet a given recommendation (Figure 1).

Almost three times as many men met ACSM/AHA^3^ and USDHHS^1^ than ACSM/AHA^1^ (73% *vs.* 27%) with a key difference being whether the required ‘dose’ of physical activity distributed adequately over the week (e.g., USDHHS^1^ represents almost exactly the same amount of recommended time as ACSM/AHA^1^ but not explicitly distributed strategically over a specified number of days).

**Figure 2:**
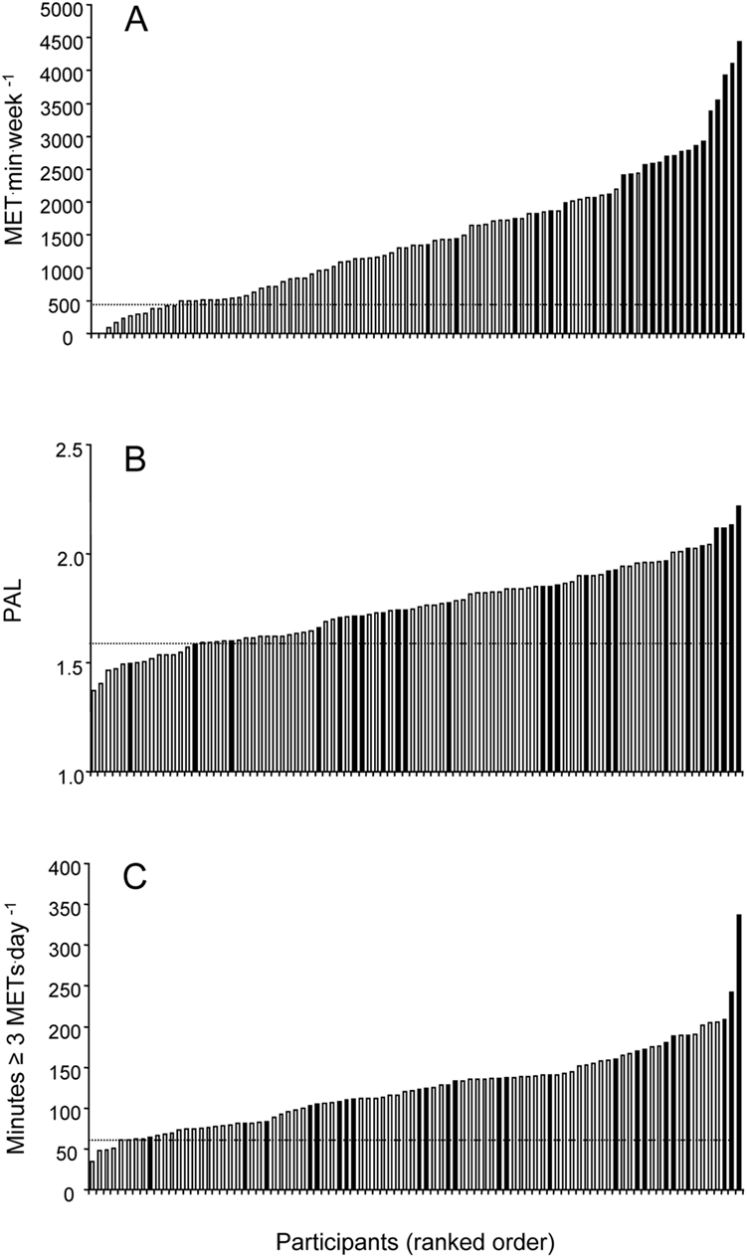
Ranked individual data for total MET^.^min per week (Figure 2a) which is equivalent to the ACSM/AHA^2^ recommendation, PAL (Figure 2b) which is the basis for the IOM^1^ recommendation, and average minutes of moderate intensity physical above 3 METs per day, which is the basis for IOM^2^ (Figure 2c). The horizontal dashed line indicates the threshold identified for each recommendation - every person above this threshold meets each respective recommendation. The shaded columns indicate where this particular subject also met the current ACSM/AHA^1^ recommendation (i.e., either 5 days of moderate activity or 3 days of vigorous activity).

Figure 2 shows ranked individual data for ACSM/AHA^2^, IOM^1^ and IOM^2^. In addition, we have superimposed upon this figure whether each subject met the current ACSM/AHA^1^ recommendation (i.e., either five days of moderate activity or three days of vigorous activity). The subject with the lowest volume of activity undertaken in bouts of 10 min who also met ACSM/AHA^1^ was 1348 MET·min·wk^−1^, although it is noteworthy that there is another subject who undertook 2442 MET·min·wk^−1^ who did not meet ACSM/AHA^1^. This discrepancy becomes more pronounced when comparing IOM^1^ or IOM^2^ to ACSM/AHA^1^. There was one person who met ACSM/AHA^1^ with a PAL of just 1.5 and who engaged in just over 60 min of moderate intensity activity per day, whereas at the other extreme there was one person with a PAL over 2.0 and several people who undertook more than 200 min of moderate intensity physical activity per day who did not meet ACSM/AHA^1^ (Figure 2b & c).

## Discussion

We examined the commonality in the classification of physical activity status between various ostensibly similar physical activity recommendations for general health [1], [3]–[6], [8]. We took into account variations in interpretation where recommendation statements were either ambiguous or contradictory; or where the same recommendation was expressed using different outcomes (e.g., time *vs*. energy). It is clear from the current analysis that using different physical activity guidelines generates large variation in physical activity status in the same individuals with very poor agreement between classifications based on different recommendations. Indeed, using different guidelines but the same raw data, anywhere between 11% and 98% of this sample of middle-aged men can be described as meeting physical activity recommendations. Only 7 men (8%) met all 12 recommendations and would therefore be unanimously classified as ‘active’ and only one man failed to meet at least one recommendation and would therefore be unanimously classified as ‘not sufficiently active’.

Over 90% of men in the present study could be variably described as either not sufficiently active or active depending on the application of different recommendations. Whilst most men in the current investigation undertook sufficient activity to meet the guidelines that recommend a specific *volume* of physical activity (assessed on either a minute-by-minute basis or in bouts of 10 min), this was not adequately *distributed* over the week to meet either the four day or five day aspect of different recommendations. It is noteworthy that a high or low volume of activity for a given individual was not an indicator of whether this person either met or did not meet a recommendation that required physical activity to be adequately distributed throughout a week. Physical activity is a complex behaviour that must be dissected and summarized in order to derive meaningful descriptors that discriminate between individuals. It may appear counterintuitive that someone who undertakes twice the volume of activity per week than another person would be defined as not sufficiently active or sedentary and judged to be failing to obtain the health-benefits of physical activity just because they do not do this activity adequately dispersed over the week. This is not wholly inconceivable, however, because an adaptive response to the last bout of activity could rely on an appropriate distribution of activity throughout the week to maintain any associated health benefit. For example, there is some evidence that exercise-induced glycaemic control and improvements in post-prandial lipaemia are relatively short-lived phenomena lasting just hours or days [23]–[25] and, as a result, the distribution of activity over a week could be important. However, it would be rather surprising that the strategic distribution of vigorous physical activity over three days or moderate physical activity over at least five days in bouts of at least 10 minutes is the minimum way to achieve this. With this in mind, there is some evidence that shorter bouts [26]–[28] and fewer days [29] are effective for at least some health outcomes.

Our results show that whether physical activity can be accumulated on a minute-by-minute basis or whether the activity has to be accumulated in bouts of 10 minutes has a major impact on whether people meet a given recommendation. This has implications for both researchers and clinical practice. Furthermore, it is noteworthy that in the UK the NICE clinical guidance for physical activity recommends that primary care practitioners should advise adults to aim for ‘30 min of moderate activity on five days a week’ [12] and there is no mention of the Chief Medical Officer's (CMO's) specific recommendation that this activity should be undertaken in bouts of at least 10 minutes [3]. Furthermore, the tool recommended in the NICE guidance for identifying inactive patients in General Practice does not assess bouts of activity nor does it assess whether the activity is distributed throughout the week [30]. These apparently trivial oversights in the interpretation of the CMO's recommendation have an enormous impact. For example, further inspection of our data reveals that 98% of men in the current study would be described as active using the NICE interpretation (30 min of moderate intensity activity greater than 3 METs on at least five days a week) whereas only 24% met the CMO's recommendation with the additional stipulation to undertake this activity in bouts of 10 min (DoH). Therefore, ostensibly small differences in recommendation, or misinterpretation of a specific recommendation, substantially affect apparent physical activity status. There are clear ramifications for applied clinical practice since primary care practitioners may inadvertently form the conclusion that some patients are meeting a specific recommendation (e.g., DoH) and encourage them to continue with their current (perhaps rather sedentary) physical activity behaviours.

Public health campaigns often promote the concept of 30 min of physical activity a day [31] although more frequently this is translated into a target of 150 min of moderate intensity physical activity a week [32]–[36]. Many guidelines appear to advocate 150 min of moderate intensity physical activity per week (ACSM/AHA^1^, ACSM/AHA^2^, USDHHS^1^). Every subject in the current analysis accumulated more than 150 min of moderate intensity physical activity per week and, moreover, they undertook more than 30 min on average per day (>210 min per week). However, this proportion falls to around three quarters of the sample if we stipulate that the 150 min of moderate intensity activity a week has to be accumulated in bouts of 10 min (ACSM/AHA^2^, USDHHS^1^) and it is noteworthy that only a quarter of the men in the current sample met the ostensibly similar target to undertake this amount of activity distributed over a certain number of days (e.g., ACSM/AHA^1^, DoH).

It is important to highlight that we have objectively monitored physical activity for almost every minute of every day, whereas in the past most investigations have only captured certain elements of physical behaviour such as walking or leisure-time activity through the use of questionnaires [37]–[41]. Even studies that use objective measurement of physical activity base their estimates on an incomplete period of observation typically around 10–14 h a day [42], [43]. It is noteworthy that even using improved instruments such as accelerometers, if two hours of activity per day are not measured then at the end of a 7-day period almost an entire day of activity will have been overlooked (14 hours of observation). If we had not included an entire day of physical activity for our sample, or if we had only obtained snapshots of certain physical activity behaviours (e.g., from questionnaires), then of course the reported amount of physical activity undertaken by our participants would appear much lower. If we do not capture the totality of physical activity then instead we have only a snapshot of physical activity behaviour. Recommendations derived from epidemiological or experimental studies based on an incomplete analysis of physical activity behaviour will lead to a relatively lower time-threshold for physical activity in relation to a given health outcome than if participants had been observed over a full 24-h cycle. This is a classic calibration or measurement error. If techniques that have been used in the past underestimate the true level of physical activity or capture only a sub-component then our analysis (that uses a better technique that monitors every minute of every day) will inevitably lead to the impression that many participants accumulate large amounts of activity. By implication, the complete observation of an individual in either research or clinical practice will generate ostensibly inflated physical activity levels and this may lead to the erroneous conclusion that physical activity behaviour is sufficient when this may not be the case in the context of the original health-related observation. Consequently, in parallel with technological advancements in measurement precision, future reincarnations of physical activity recommendations should be based on a rigorous re-evaluation of the associations between *total* physical activity and various health outcomes.

It is not possible based on the current analysis to determine if one recommendation is superior to another. However, it is clearly inappropriate that an individual who is described as ‘highly active’ by one physical activity descriptor could be labelled ‘not sufficiently active’ according to another. Physical activity is complex and a single ‘descriptor’ is unlikely to capture the key dimensions that unambiguously characterize a given individual or population. Future research should seek to apply better tools for the assessment of physical activity and generate a clearer understanding of the biological basis for physical activity recommendations through classical ‘dose-response’ experiments. The long-term target should not be a single one-size-fits-all recommendation based on one dimension of physical activity but instead perhaps a smorgasbord of physical activity ‘options’ that have been titrated against measurable health outcomes in defined populations. As future physical activity recommendations evolve and our ability to monitor behaviour improves, it will probably become more important to make recommendations that target the multiple dimensions of physical activity behaviour and it will probably also be important to use a more continuous scale of measurement.

One limitation to the current investigation could be the instrument used to assess physical activity. This is the first time that a technique with sufficient precision to assess activity energy expenditure as well as patterns of physical activity over continuous unbroken periods of time has been available to allow such a comparison. Importantly, whilst this is an excellent instrument [14]–[17], it is important to highlight that any small error will have little effect on the findings since the actual output is the same for all comparisons and the reported differences are mostly due to the way in which the output is dissected post-measurement. For example, the Schofield equation tends to overestimate basal metabolic rate in men [44]. Any overestimation of basal metabolic rate would clearly result in a systematic underestimation of physical activity energy expenditure expressed in METs (and hence time spent above the 3 MET threshold). However, the standard error of prediction for the Schofield equation in men is less than 0.5 MJ/day, which translates to less than 0.1 kcal/min [44]. We believe that whilst such small differences between measured and estimated resting metabolic rate could potentially be important if we were to use this information in clinical practice, this does not affect our analysis in the present study since this is a within-subject comparison based on the analysis of the same raw data. It is noteworthy that the middle-aged men included in the present investigation appear to have similar levels of physical activity to the UK population using the Department of Health physical activity recommendation [3] and had the same average PAL as reported in the Institute of Medicine doubly-labelled water database for men of this age [4]; although these men are not necessarily representative of the general population. Our sample represents a heterogeneous sample of middle-aged men and we do not know whether our findings would also apply to segments of the population who are either more or less active or who accumulate their activity in different ways. Furthermore, we did not include women in our sample and therefore we do not know if the commonality between recommendations is better, similar or worse for women or, indeed, for younger or older men.

In conclusion, we have shown that physical activity status varies enormously depending on the physical activity recommendation that is applied and even ostensibly small differences have a major impact on classification. For nine out of ten men in the present study, the answer to the question ‘Am I doing enough physical activity for my health?’, appears to be simultaneously ‘yes’, ‘no’ and ‘it depends’. Either the effective dose or prescription that underlies each of the various physical activity recommendations is different or each recommendation is seeking the same prescriptive outcome but with variable success. This problem is further compounded by the suggested use of tools in applied clinical practice that do not assess critical aspects of the prescription (e.g., GPPAQ in the UK). There have been recent calls and efforts to evaluate and improve diagnostic tests and assays in applied clinical practice [45]. The same rigour should be applied to tests of physical activity status in order to avoid the present conflict and confusion, which may compound the “second gap” of the translation of research evidence into practice identified in the Cooksey report [46].
